# A Hierarchical Bayesian Approach to Ecological Count Data: A Flexible Tool for Ecologists

**DOI:** 10.1371/journal.pone.0026785

**Published:** 2011-11-21

**Authors:** James A. Fordyce, Zachariah Gompert, Matthew L. Forister, Chris C. Nice

**Affiliations:** 1 Department of Ecology and Evolutionary Biology, University of Tennessee, Knoxville, Tennessee, United States of America; 2 Department of Botany, Program in Ecology, University of Wyoming, Laramie, Wyoming, United States of America; 3 Department of Biology, University of Nevada, Reno, Nevada, United States of America; 4 Department of Biology, Population and Conservation Biology Program, Texas State University, San Marcos, Texas, United States of America; Universita' del Piemonte Orientale, Italy

## Abstract

Many ecological studies use the analysis of count data to arrive at biologically meaningful inferences. Here, we introduce a hierarchical Bayesian approach to count data. This approach has the advantage over traditional approaches in that it directly estimates the parameters of interest at both the individual-level and population-level, appropriately models uncertainty, and allows for comparisons among models, including those that exceed the complexity of many traditional approaches, such as ANOVA or non-parametric analogs. As an example, we apply this method to oviposition preference data for butterflies in the genus *Lycaeides*. Using this method, we estimate the parameters that describe preference for each population, compare the preference hierarchies among populations, and explore various models that group populations that share the same preference hierarchy.

## Introduction

Count data is frequently used in studies of ecology, behavior, and evolutionary biology. Behavioral count data might include the number of approaches to a particular mate phenotype [1], the number of times aggressive displays are observed [2], or the number of eggs laid on various oviposition substrates [3]. Ecological data might include the number of seeds germinated [4], or the number of parasitized individuals [5]. Evolutionary data might include the number of offspring in a particular ecological arena [6] or, conversely, the number of deaths [7]. Statistical analyses of count data are then used to guide biologically relevant inferences. A battery of methods have been developed to analyze count data [8], [9], [10].

Frequently, these statistical methods model the data in the form of analysis of variance (ANOVA), or use methods often regarded as their non-parametric equivalents. The *p* values provided by these tests are then used in a traditional sense to guide statistical inference. For example, item A might be significantly chosen more often than item B based upon an *a prioi* determined 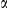 value, usually 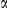 = 0.05. However, often the parameter of interest is not directly modeled when carrying out such analyses. For example, imagine an experiment with 20 replicates where two host plants are provided to an herbivore and the number of eggs laid on each plant (count data) is the response variable. One might analyze these data as a paired *t*-test, where each pair is the pair of plants in each experimental arena. Here, the test is not directly estimating the strength of preference (the true parameter of interest) for each plant at the individual or population level, rather it is examining whether the mean difference in preference is different from zero. Instead, the strength of preference is often estimated as a proportion of eggs laid on each plant over the experiment, or the mean of the proportions across replicates, in a *post-hoc* manner. Some notable potential problems with this approach include that the statistical analysis is largely independent of the parameters of interest and that the statistical analysis itself does not directly incorporate uncertainty at the level of the individual replicate. Often, ANOVA on proportions is implemented on such experiments. Although weighting schemes can be applied, this approach does not generally account for uncertainty around those proportions for each replicate. That is, it will not directly account for differences in the total counts per replicate (such as might occur if there is substantial variation in the number of observations per replicate), or the uncertainty around the proportions calculated for each of those replicates. Additionally, proportional data will frequently violate the assumption of normality for the response variable even after the commonly used arcsin square root transformation is applied [11], [12]. Non-parametric, or rank-based, methods can be used to overcome some of the problems associated with parametric analyses. Most of these methods are based on rankings of observations within replicate, such as the commonly used Friedman test. However, this test also fails to account for differential information provided in each replicate (i.e., among replicate variation in the total number of observations). The Quade test was proposed as an alternate to the Friedman test to account for these differences [10]. Here, not only are the choices within replicate ranked, but also the total number of observations among replicates are ranked. Each replicate is thus “ranked” based on the amount of information provided. For example, a replicate with 50 total eggs laid is weighted more heavily in the analysis compared to a replicate with only 10 eggs. However, these methods also fail to directly estimate the parameter of interest, the parameter that describes preference in the above example.

Here, we describe an alternate approach to count data; a hierarchical Bayesian approach. This approach has the advantage that it directly estimates the parameters of interest (those that describe preference) and appropriately models uncertainty. Further, this approach also provides a framework to compare the parameter estimates among *a priori* defined groups (e.g., populations, families, environments, etc.). As an example, we apply this method to oviposition preference data for butterflies of the genus *Lycaeides* (Lepidopetera: Lycaenidae) from various populations in western North America. Our goal is not to delve deeply into the evolution of host plant preference in this group, rather to use this experimental data as an exemplar of how this hierarchical Bayesian approach can be used for this and similar types experimental data.

## Materials and Methods

### Hierarchical Bayesian Model for Count Data

The count data for each individual within a population is modeled as a hierarchical Bayesian model. This approach is applicable to any data that are recorded as counts (i.e., integers), and individuals need not necessarily be the lowest level in the hierarchy. For example, cafeteria experiments where multiple resources are available in a field setting, or pooled choice experiments where multiple individuals are confined to an experimental arena, can apply this method. Additionally, this method need not be restricted to choice data, and might include number of individuals dying under different conditions, number of lesions following infection by various pathogens, etc. The only requirement beyond count data, is that the investigator is explicitly aware of what each hierarchical level describes (i.e., response at the level of individual, cage, feeding station, sample, etc.).

For simplicity (and consistency with the example below), the model is described as oviposition preference data (i.e., the number of eggs an individual female laid on each plant provided in the oviposition choice arena) obtained from multiple females (i.e., experimental replicates) to estimate the population level preference. The response for each individuals' choices are modeled as a multinomial distribution with a unique set of parameters that reflect the preference for that individual, thus, for each individual, we model 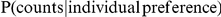. This gives rise to the first level likelihood model,
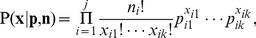
(1)which is the product of 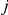 multinomial distributions, where 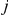 is the number of individuals (i.e., experimental replicates). **x** is the count data for all individuals among the 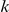 number of plants to choose among. **n** is a vector of counts, or number, of eggs laid on each plant by each individual. 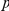 are the probabilities (contained within the vector, 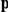) of laying an egg on each plant for each individual. Because we are interested in estimating population-level preference, in addition to individual-level preference, we assume that this vector of parameters describing each individual's preference for each plant is drawn from a Dirichlet distribution, the continuous analog of a multinomial distribution, describing the prior probability of preferences that characterize the population. This prior probability is not specified for the analysis, rather it is estimated from the data. Thus, we model 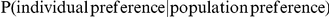, the probability of an individual's preference given the population-level preference. This gives rise to a conditional prior for individual preferences, a Dirichlet,
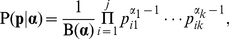
(2)where the 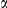 parameter is decomposed into two elements that describe the mean expected values, 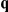, a vector for which all elements share the same scaler parameter (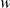) that describes the variance. Thus, it enables the estimation of the mean and variance of the Dirichlet distribution separately. For the parameter vector 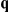, we assume an uninformative Dirichlet prior (i.e., Dirichlet (1,1, .,1)), and for 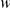 we assign a uniform prior (i.e., 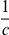, where 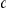 is the upper bounds of the uniform distribution). However, alternate prior distributions may be assigned if deemed appropriate based upon knowledge of the experimental system of interest. Thus, our conditional prior for individual preference is
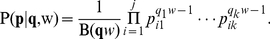
(3)This specification yields the following hierarchical Bayesian model,
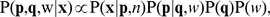
(4)or rewritten after substituting mathematical equations for the probability statements,
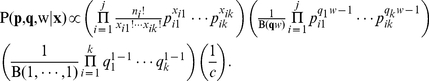
(5)The posterior probability of the individual preferences is proportional to the likelihood function describing the probability of the count data, multiplied by the conditional prior probability of individual preferences, multiplied by the prior probability of the mean of individual preferences and the prior probability of the variance in individual preferences. The likelihood function is used to calculate the probability of the multinomial distribution of eggs laid on each plant (**x**) given the vector of probabilities for each individual laying an egg on each plant (**p**) and the vector of counts for each individual (**n**). This is multiplied by three prior probabilities: The conditional prior describes the probability of each individual laying an egg on each plant (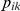) given the vector of expected values (**q**) and scaler parameter (*w*). The second prior is the probability of the vector of expected values (**q**), and the last term is the prior probability of the scaler parameter (*w*).

**Figure 1:**
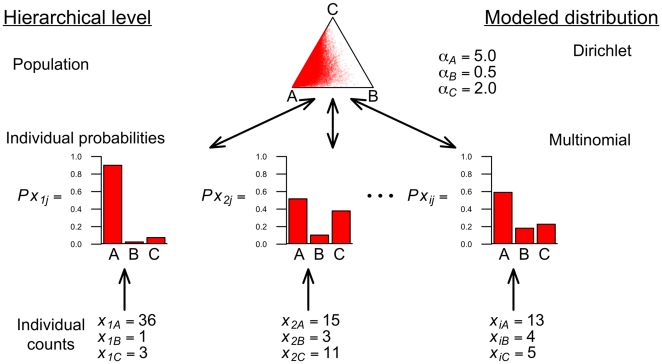
Schematic of hierarchical Bayesian model for count data. Individual count data inform the parameters for each individual's multinomial parameters. The multinomial parameters for all individuals inform the population level preference modeled as a Dirichlet. This population-level preference is shown as a ternary diagram (a triangle plot). The population-level preference, in turn, informs the most likely individual multinomial parameters given the population preference. Thus, at each MCMC step information is passed from the individual preferences to the population preference, and *vice versa*. Note that the 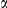
's and 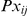 are not fixed for the analysis. The role of the hyperpriors on *w* and *q* are not depicted in the figure.

Parameters are estimated using a Markov chain Monte Carlo (MCMC) where, at each step in the chain, individual preferences (based on the multinomial distribution for each individual) inform the population-level preference. In turn, the population-level preference (based on the parameters of the Dirichlet distribution), inform the probability of each individual's multinomial parameters (Figure 1). Thus, the analysis simultaneously estimates individual-level preferences and the population-level preference. Individual-level preference can be examined based upon the posteriors for each individual's preference parameters, or by examination of the variance term from the Dirichlet distribution (*w*, which is inversely proportional to the variance). For experimental designs where there are two possible choices, the model simplifies to a special case of multinomial and Dirichlet distributions where individual-level preferences are modeled as binomial distributions with parameter 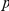 drawn from a common, population-level, beta distribution. At the completion of the MCMC run, we are provided with posterior probability distributions for the preference of each individual, as well as the posterior probability distribution for the population preference as a whole. Analogous to a traditional *post-hoc* test, we can examine the “significance” of differences in preference among the choices by examining the proportion of times a given pairwise comparison is greater or less than the other choice at each step in the post-burnin MCMC. That is, for example, if item A has a higher ranked estimated population-level parameter value (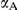; a measure of the strength of preference for item A compared to the estimated preference for item B (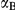)) across 99% of the post-burnin MCMC steps, we can conclude that the probability that the preference for item B is equal to, or greater than the preference for item A is 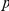 = 0.01. Although not required for interpreting the results of the hierarchical Bayesian model presented here, this pairwise probability method provides a familiar framework for interpreting significant differences among choices offered to the population.

### Model Selection and Performance

Deviance information Criterion (DIC) can be used to compare models with alternate population groupings [13]. Here, we used DIC to examine whether groups of populations could be modeled as drawing preferences from a common, population-level distribution, where all preferences were equal compared to a population-level distribution where preference differed among possible choices. Simply, whether preference is the same among possible choices, or whether it differs. DIC is analogous to Akaike information criterion (AIC) [14] and is well suited for model comparison in a Bayesian framework when posterior distributions are approximated via MCMC [13]. The deviance of a model is,
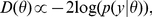
(6)where 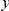 are the data, 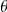 are the model parameters, and 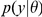 is the product of the likelihood and conditional prior (Eqns. 1 and 3). DIC is calculated as,
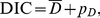
(7)where 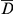 is the posterior expectation of the deviance and 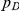 is the effective number of parameters calculated as,
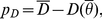
(8)or the expected deviance minus the deviance examined at each posterior expectation. For an in depth discussion of DIC, see Spiegelhalter 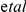. [13]. Similar to AIC, models with lower DIC values have greater support [14]. There is no general consensus on how large the difference in DIC values (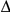
DIC) among models needs to be before a model, or models, should be excluded for consideration as those that best fit the data; however, Spiegelhalter 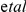. [13] suggested that important differences can be interpreted as with AIC as suggested by Burnham and Anderson [14], where models within 2 units of the ‘best’ model deserve consideration, whereas others have suggested up to 10 DIC units.

To examine whether this approach might be prone to favoring an over-parameterized model, we compared the performance of the hierarchical Bayesian modeling approach proposed here to three commonly used conventional approaches; the Friedman test and the Quade test (both commonly used non-parametric methods [10]), and ANOVA on arcsin square root transformation of proportions, using simulated data. Each simulated data set consisted of 20 replicates with 3 choices each, where each replicate might be considered an individual female in a choice arena with 3 possible host plants. The total number of choices made for each replicate, or the number of eggs laid by each female, was randomly drawn from a uniform distribution bounded at 5 and 40 rounded to the nearest integer. Individual choices for each replicate were random draws from a multinomial distribution with parameter values drawn from a population-level Dirichlet distribution with 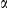 parameters equal to 1. That is, we simulated choices made by individuals drawn from a population with no preference, the null expectation if there is no preference among each of the possible choices. In total, 1000 simulated data sets were examined.

### Study system and oviposition preference experiments

*Lycaeides* is a holarctic genus with at least five nominally recognized species in North America: *L. anna*, *L. idas*, *L. melissa*, *L. samuelis* [15], and a recently described homoploid hybrid species that occupies alpine habitats in the Sierra Nevada [16]. The group has received considerable attention as a model system for studies on local adaptation, ecological speciation, and hybridization [1], [16], [17], [18], [19], [20], [21], [6], [22], [23], [24], [25]. One important factor for the maintenance of variation among populations is host plant preference and fidelity (*sensu* Feder [26]). Previous studies have shown that the strength of preference for various host plant species varies among populations [3], [16], [6]. We examine host plant preference variation among *Lycaeides* populations using the hierarchical Bayesian model on experimental oviposition preference data that was originally analyzed as the proportion of eggs laid on the natal host in Gompert *et al.* [16]. These populations, hereafter referred to as *focal populations*, include seven localities. All of these populations use perennial legumes as larval host plants. Gardnerville, NV and Verdi, NV are nominally *L. melissa* and use agricultural and feral alfalfa (*Medicago sativa*) as their primary host plant [3], [16]. Leek Springs, CA, Trap Creek, CA, and Yuba Gap, CA are nominally *L. anna*. Both the Trap Creek and Yuba Gap populations occupy boggy habitats and use *Lotus nevadensis*. Leek Springs uses *Lupinus polyphyllus* as a host plant. Populations at Carson Pass, CA and Mt. Rose, NV occur above tree line in the Sierra Nevada and use *Astragalus whitneyi* as a host plant. These alpine populations have previously been shown to be a distinct species of hybrid origin [16].

Oviposition preference was examined by confining single, wild caught females in an oviposition arena that included four possible plant species to choose among; *A. whitneyi*, *L. nevadensis*, *L. polyphyllus*, and *M. sativa*. Each oviposition arena was a plastic container (diameter = 11.5 cm, height = 13 cm) containing the four host plant choices with spun polyester mesh covering the top. Four small holes at the bottom of each cup allowed for the stem of each plant to extend into a water reservoir secured to the bottom of each arena. After 48 hours of confinement in the arena, the number of eggs laid on each host plant species was recorded as a measure of each female's preference among host plant choices provided (see Gompert *et al.* [16] for more details on experimental design). We used DIC to determine if a given population's preference is best modeled as an equal preference for all host plants provided (i.e., no preference among choices), or if a model that has separate preference parameters for each host plant best fits the data (i.e., variation in preference among choices). The strength of preference for each host plant species in each population was assessed by examining the posterior distributions for each of the parameter estimates, and by examining the pair-wise proportion of times that a given host plant had a preference parameter of greater value compared to another plant species at each step of the MCMC. Further, we examined various population grouping schemes to determine which populations might best be modeled as sharing the same preference parameters across these possible host plant species.

We similarly examined variation in preference for four other populations of *Lycaeides* (Big Pine, CA, Cave Lake, CA, Eagle Peak, CA, and White Mountains, CA), with special attention paid to the strength of preference for *A. whitneyi*. The experimental approach here was similar to that described above, except here three females were confined simultaneously to each oviposition arena. Assuming that the combined preference of the three females in each cage is a sample of the population-level preference overall, we are still able to estimate the population-level preference in this statistical modeling framework. Here, individual-level preference cannot be estimated because the lowest hierarchical level of preference is at the level of arena; however, the population-level parameters describing preference can be estimated based upon the arena-level preference estimates.

## Results and Discussion

**Figure 2:**
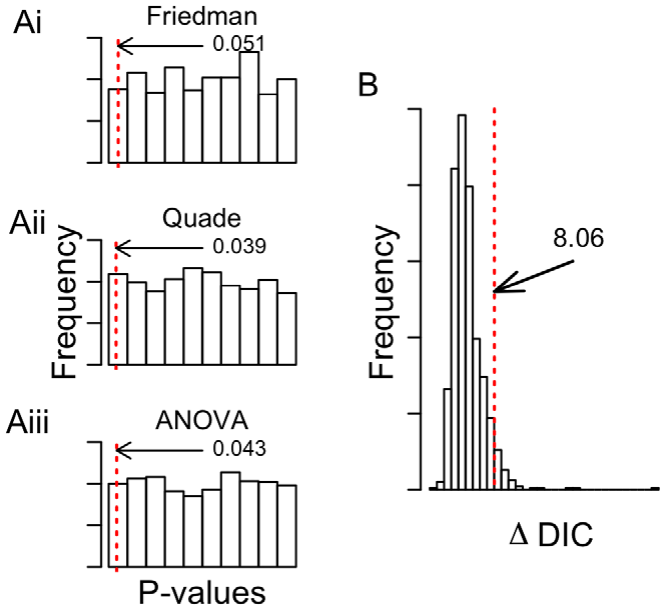
Simulations examining performance. A) Performance of conventional methods for analyzing count/preference data. Red hatched line indicates the 0.05 quantile of p-values for 1000 data sets simulated under the null model of no preference. Numbers are the *p*-values of 0.05 quantile. Methods examined were the (i) Friedman test, (ii) the Quade test, and (iii) ANOVA on arcsin square root transformed proportions. B) Distribution of 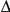
DIC values for models with equal preference versus models with different preferences for each item. Red hatched line indicates the 0.95 quantile of 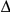
DIC values.

As would be predicted, the distribution of p-values obtained from the Friedman test, Quade test, and ANOVA on the simulated data sets were largely uniform, with the 0.05 quantile of p-values near 0.05 (Figure 2Ai–iii). Under these simulations, the 0.05 quantile of 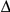
DIC was near 8 (Figure 2B), indicating that blindly accepting a 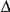
DIC of 2 or greater for model selection as recommended by Spiegelhalter 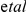. [13] might favor an over-parameterized model. As with all model selection tools, DIC should be treated as a subjective method for comparing the performance of competing models.

**Figure 3:**
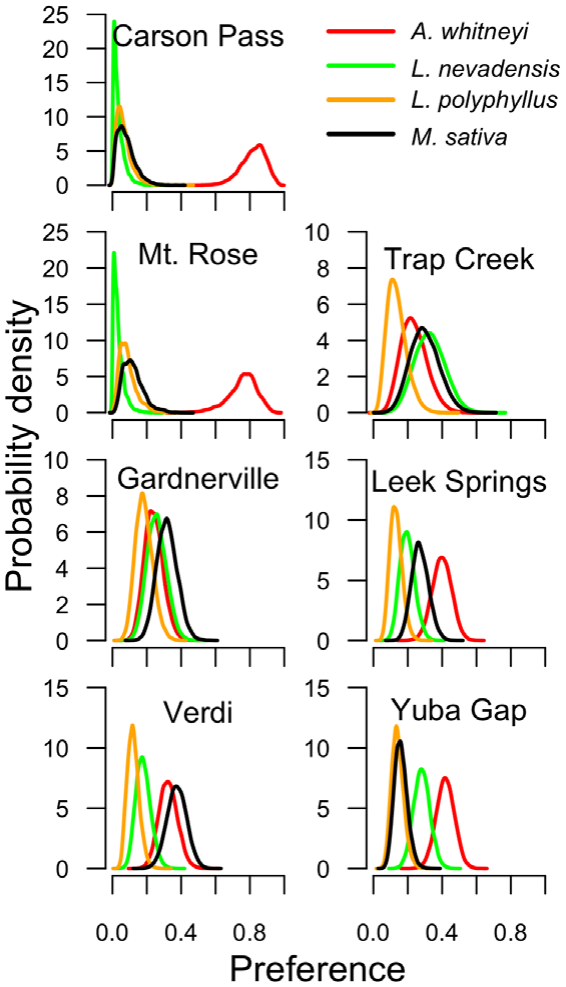
Host plant preferences for focal populations. Colored curves indicate posterior density for population-level preference for four host plant species. Posterior densities estimated from 40000 MCMC steps following a burnin of 10000 generations.

**Figure 4:**
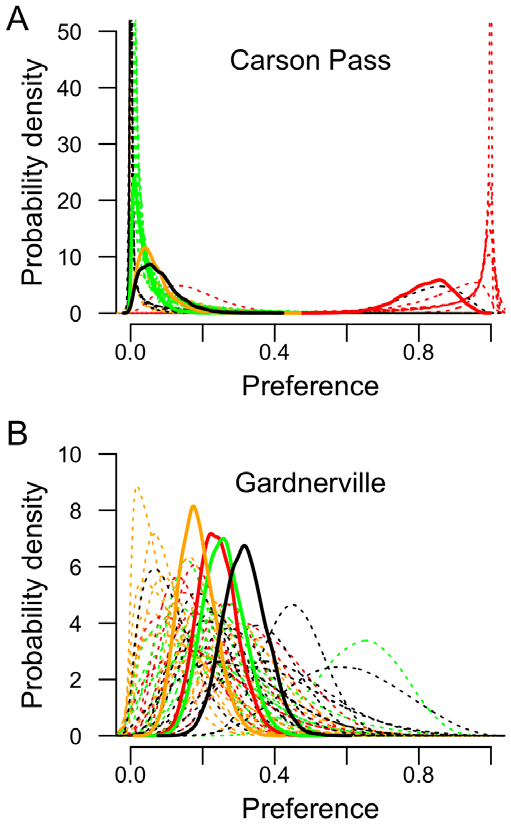
Population and individual preferences. Population-level preferences (solid lines) and individual-level preferences (dotted lines) for each of the four host plants. Colors for each plant as in figure 3. Populations presented are A) Carson Pass and B) Gardnerville. Posterior densities estimated from 40000 MCMC steps following a burnin of 10000 generations.

The median number of eggs laid by females in the oviposition arenas was 9.5 eggs, with a range of 1 to 40. There was evidence for varying degrees of strength of host plant preference among populations (Figure 3). The preference for the natal host plant varied among populations, with most populations showing a higher preference for *A. whitneyi* compared to the other host plants offered. With the possible exception of the *L. melissa* population from Gardnerville, NV, an unconstrained model was favored over a constrained model based on DIC scores for each population (Table 1). Figure 4 illustrates both the estimated population preferences, as well as the estimates of all individual preferences in the sample, for the populations at Carson Pass, CA and Gardnerville, NV. Thus, there is evidence that a preference hierarchy exists for most, if not all, of the populations examined here. As observed for these populations previously [3], [16], the strongest preference was detected for the alpine, hybrid species at Carson Pass and Mt. Rose, which showed extremely high preference for their natal host plant, *A. whitneyi* (pairwise post-burnin comparisons; *p* 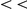
0.01 for all comparisons between *A. whitneyi* and the other three test plant species). The *L. anna* populations showed less preference for each of their respective natal host plants. The *L. polyphyllus*-feeding population at Leek Springs showed low preference for their natal host plant, with their strongest preference for *A. whitneyi*. The *L. nevadensis*-feeding populations at Yuba Gap and Trap Creek showed mixed degrees of strength in preference. Yuba Gap showed an overall preference for *A. whitneyi* and *L. nevadensis*, whereas Trap Creek showed less variation in preference for the host plants presented. Though it should be noted that the ability to detect differences in preference might be a consequence of insufficient replication to adequately estimate the population-level preference for Trap Creek. The strength of preference also varied between the two *L. melissa* populations using *M. sativa*. The population at Verdi showed stronger preference for both *M. sativa* and *A. whitneyi* compared to the other two plant species offered, whereas the population at Gardnerville showed little evidence of preference for any of the four plants offered. Comparisons among models where population preferences were contrained among groups indicated that, for this data, the best fit model for preference is one that fits well along taxonomic boundaries (Table 2). More important, it is clear that a model constraining the preference parameters to be the same across all populations is inappropriate.

**Table 1:** Populations and DIC values for constrained and unconstrained preference.

Population	Natal host plant	N	Constrained DIC	Unconstrained DIC
Carson Pass, CA	*A. whitneyi*	12	−240.85	−5162.92
Mt. Rose, NV	*A. whitneyi*	13	−218.15	−929.68
Gardnerville, NV	*M. sativa*	15	67.71	63.03
Verdi, NV	*M. sativa*	14	97.06	79.01
Leek Springs, CA	*L. polyphyllus*	8	107.74	93.15
Trap Creek, CA	*L. nevadensis*	14	6.65	−17.59
Yuba Gap, CA	*L. nevadensis*	13	101.83	87.41

Constrained refers to models where preference for each plant is equal. Unconstrained refers to models where preferences are permitted to vary among host plants. N is the number of replicates for each population.

**Table 2:** DIC comparisons among grouping schemes for host plant preferences.

Grouping	DIC
(CP,MR,GV,VE,LS,TC,YG)	137.61
(CP)(MR)(GV)(VE)(LS)(TC)(YG)	−3382.07
(CP,MR)(GV,VE)(LS)(TC)(YG)	−5416.32
(CP,MR)(GV)(VE)(LS)(TC)(YG)	−5530.88
(CP,MR)(GV,VE)(TC)(LS,YG)	−5597.75
(CP,MR)(GV,VE)(LS,TC,YG)	−6085.99

Parenthetical groups constrained to have same preference parameters in the model. DIC values based on 40000 MCMC steps following a burnin of 10000 generations. Abbreviations are as follows: CP, Carson Pass; GV, Gardnerville; LS, Leek Springs; MR, Mt. Rose; TC, Trap Creek; VE, Verdi; YG, Yuba Gap.

Overall, there was a general trend for most populations to favor *A. whitneyi* in the experiments. This preference for *A. whitneyi* over other host plant species was also detected in other populations of *Lycaeides*, including populations where the natal host plant is not *Astragalus* (Table 3). The population at Big Pine, CA, nominally called *L. melissa inyoensis*, is associated with marsh habitat in the Owens Valley and feeds on *Glycyrrhiza lepidota* as larvae. However, females from this population preferred *A. letiginosus* over their natal host plant. Similarly, the Cave Lake, CA population, nominally *L. idas ricei*, showed strong preference for *A. whitneyi* over *M. sativa* and *Vicia americana*. This population is found in wet habitats and is associated with *V. americana*, though they might also use *L. polyphyllus* as a larval host plant (pers. obs.). Two other populations at Eagle Peak, CA and White Mountains, CA occupy alpine habitat and, similar to the populations at Carson Pass and Mt. Rose, showed strong preference for *A. whitneyi*. The populations at Eagle Peak and White Mountains are also likely of hybrid origin [21] and share many traits with Carson Pass and Mt. Rose, including intermediate egg [19] and genitalic morphology [20] and low egg adhesion to the host plant [17]. These alpine populations consistently showed strong preference for their natal *Astragalus* host plant. In fact, in an experiment where females from Carson Pass were introduced to an arena where only *L. polyphyllus*, *L. nevadensis*, and *M. sativa* were available, they laid 77% fewer eggs compared to females in arenas where *A. whitneyi* was present (unpaired *t*-test, *t* = 2.815, d.f. = 18, *p* = 0.01). Interestingly, the females in arenas with *A. whitneyi* absent overwhelmingly favored *L. nevadensis* (preference and 95% credible interval: 0.66 (0.40, 0.84)) over the other plants offered, suggesting that a preference hierarchy does exist even in these populations with extremely high natal host plant preference. Despite having egg and genitalic morphology that is intermediate between the putative parental species, *L. melissa* and *L. anna*, and a genome that is a mosaic of the parental genomes, the alpine associated homoploid hybrid species showed extremly high preference for *A. whitneyi*. These populations also have the unique trait of lack of egg adhesion (i.e., the eggs fall of the plant shortly after they are laid) which likely serves as an adaptation to seasonal above ground senescence of *A. whitneyi* and strong winds in the alpine habitat [17]. The strength of host plant preference and lack of egg adhesion have been suggested as a possible transgressive trait for this hybrid species [16].

**Table 3:** Non-focal population summary of preference for *Astragalus* and natal host plant, and DIC scores for constrained and non-constrained models.

Population	Test plants	N	Preference: *Astragalus*	Preference: Natal	Constrained DIC	Unconstrained DIC
Big Pine, CA	*A.l.*, G.l.*, *M.s.*	11	0.57 (0.37, 0.73)	0.17 (0.07, 0.32)	−4.04	−11.60
Cave Lake, CA	*A.w.*, *M.s.*, *V.a.*	6	0.62 (0.44, 0.78)		5.34	−44.36
Eagle Peak, CA	A.w*.,*M.s*,*V.a.*	10	0.72 (0.56, 0.87)		−22.31	−100.63
White Mts., CA	A.w*.,*M.s*,*G.l*	15	0.64 (0.44, 0.78)		−13.06	−53.16

Host plant abbreviations are as follows: *A.l.*, *Astragalus letiginosus*; *A.w.*, *A. whitneyi*; *G.l.*, *Glycyrrhiza lepidota*; *M.s.*, *Medicago sativa*; *V.a.*,*Vicia americana*. Natal plant for Cave Lake population is not definitively known, however, it is not *A. whitneyi* and is most likely *L. polyphyllus*. Constrained model is one where preference for all plants is equal, whereas the unconstrained is one where preference is permitted to vary across host plants. DIC scores based on 40000 MCMC generations following a 10000 generation burnin.

The hierarchical Bayesian approach described herein is a flexible tool for count data. It provides parameter estimates that directly address the biological hypotheses; in the present case, the strength of host plant preference across ecologically varied populations of *Lycaeides*. These estimates include not only population-level preferences, but also individual-level preferences. Variation in individual-level preference can be examined directly from the posteriors for each individual or by interpreting the variance term associated with the Dirichlet distribution. The ability to obtain this information is unique to this analytical approach compared to traditional methods. For example, if two choices are available and the population-level preference for an item is 0.5, the approach presented here will allow investigators to determine whether this population-level preference is the result of no preference for all individuals (high values for *w*, where most individual preferences are near 0.5) or, alternatively, if individuals have clear preference for either of the two choices (low values for *w* where most individual preferences for a given item are near 0 or 1). This approach also permits one to compare among various models. For *Lycaeides*, this included comparing models with a single preference parameter value for all host plants against a model where preference was permitted to vary among host plants. Further, it allowed for comparisons among various population grouping schemes, indicating which populations show similar preferences; or, more precisely, which populations are best modeled as sharing the same preference parameters. This approach is not restricted to preference data and should be broadly applicable to data recorded as counts. Implementation of this approach can be accomplished in the R statistical computing language environment [27] using the package *bayespref* (see Supporting Information S1).

## Supporting Information

Supporting Information S1 **An introduction to** ***bayespref*** **: a tutorial.**
(PDF)
