# Supplementary material for: A Hierarchical Bayesian Approach to Ecological Count Data: A Flexible Tool for Ecologists
Source: PLoS One. 2011 Nov 21;6(11):e26785. doi: 10.1371/journal.pone.0026785 (PMC3221656; doi:10.1371/journal.pone.0026785)
Supplement: Supporting Information S1 — An introduction to bayespref : a tutorial. (PDF) [file pone.0026785.s001.pdf]

An introduction to *BayesPref*

The package *BayesPref* will implement a hierarchical Bayesian analysis of count data.

Data format:
BayesPref requires that data be provided as an object class matrix. The data provided in "YGGVdata" is oviposition preference data for *Lycaeides* populations from Yuba Gap, CA and Gardnerville, NV. Here, the populations have been coded numerically. That is, Yuba Gap is population 1 and Gardnerville is population 2. The abbreviations for plant species are as follows: Ast, *Astragalus whitneyi*, Lotus, *Lotus nevedensis*, Lupine, *Lupinus polyphyllus*, and Medicago, *Medicago sativa.*

```
YGGVdata
```

Pop	Ast	Lotus	Lupine	Medicago
1	12	4	2	4
1	0	2	1	0
1	6	8	13	9
1	6	7	1	0
1	21	13	1	1
1	10	8	5	1
1	17	4	0	2
1	7	0	2	0
1	6	3	6	4
1	12	14	2	3
1	2	0	0	2
1	2	8	1	11
1	3	0	0	1
2	1	0	0	2
2	2	1	5	3
2	2	4	6	3
2	0	1	0	0
2	0	0	0	1
2	6	6	5	3
2	2	15	1	0
2	0	0	0	6
2	0	0	1	0
2	6	3	0	6
2	0	0	0	1
2	5	4	5	14
2	6	3	1	2

Note: If only one population is to be examined, the column indicating populations is not required (see below).

BayesPref analysis:
The basic hierarchical Bayesian analysis of this data can be accomplished by first converting the object containing the data to a matrix:

```
YGGV<-as.matrix(YGGV)
```

Then using the function **bayesPref**:

```
YGGVpref<-bayesPref(pData=YGGV,mcmcL=5000,pops=TRUE,dicburn=1000)
```

pData indicates the matrix of count data, mcmcL the number of steps in the MCMC. pops=TRUE (the default) indicates that the first column is the population (or experiment) identifier. If only one population is present, this column is not required and the arguement pops=FALSE should be included. The argument dicburn=100 indicates the length of the burnin for the calculated DIC score, which can be useful for comparing models.

The output, here assigned to object YGGVpref, is a list that includes the individual-level preferences, population-level preferences, population variance parameter, the likelihood, and the probability of the model at each step in the MCMC, as well as the DIC score for the model.

```
> names(YGGVpref[[1]])
[1] "IndPref"    "PopPref"    "PopVar"     "likelihood" "pMD"        "dic"
```

The defaults result in a relatively short MCMC and it is recommended that the chain (and burn in for dic) be larger. The mixing of the chains can be examined by observing the population preference parameter for a given plant across the MCMC. For example, the MCMC mixing for the Gardnerville population (population 2) can be observed as follows:

```
plot(YGGVpref[[2]]$PopPref[1,],xlab="MCMC
step",ylab="PopPref")
```

The plots below show the chain mixing for Yuba Gap. The top plot shows a well mixing chain. The bottom plot shows a poorly mixing chain. Chain mixing was affected by the argument dirvar in the **bayesPref** function. The dirvar argument is a multiplier that affects the proposal distance in the MCMC. Decreasing the value increases the proposal distance. The top plot is using the default (dirvar=2), the bottom plot used dirvar=0.5.

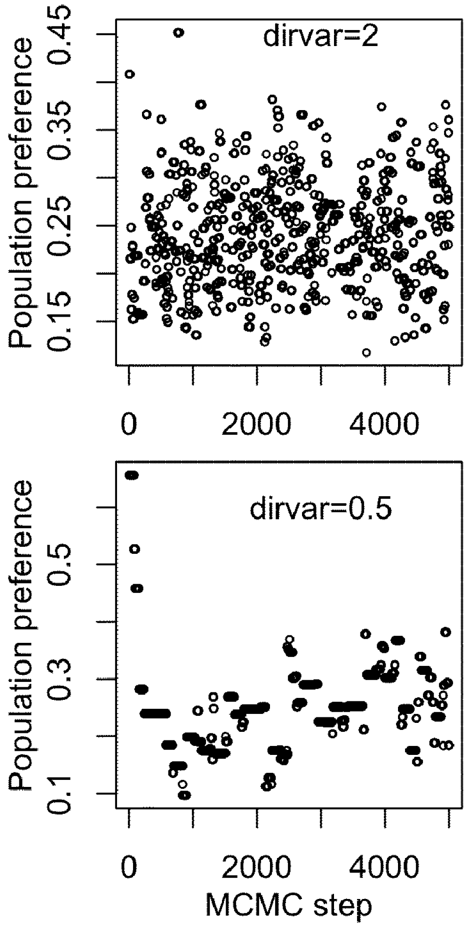

Once the user is satisfied with the mixing of the MCMC, the user can examine the parameter estimates provided by the analysis. The median parameter value and the credibility intervals for individuals and the population can by obtain with the function **credibleIntervals**.

```
credibleIntervals(prefres=YGGVpref[[1]],burn=1000,interval=0.95)
```

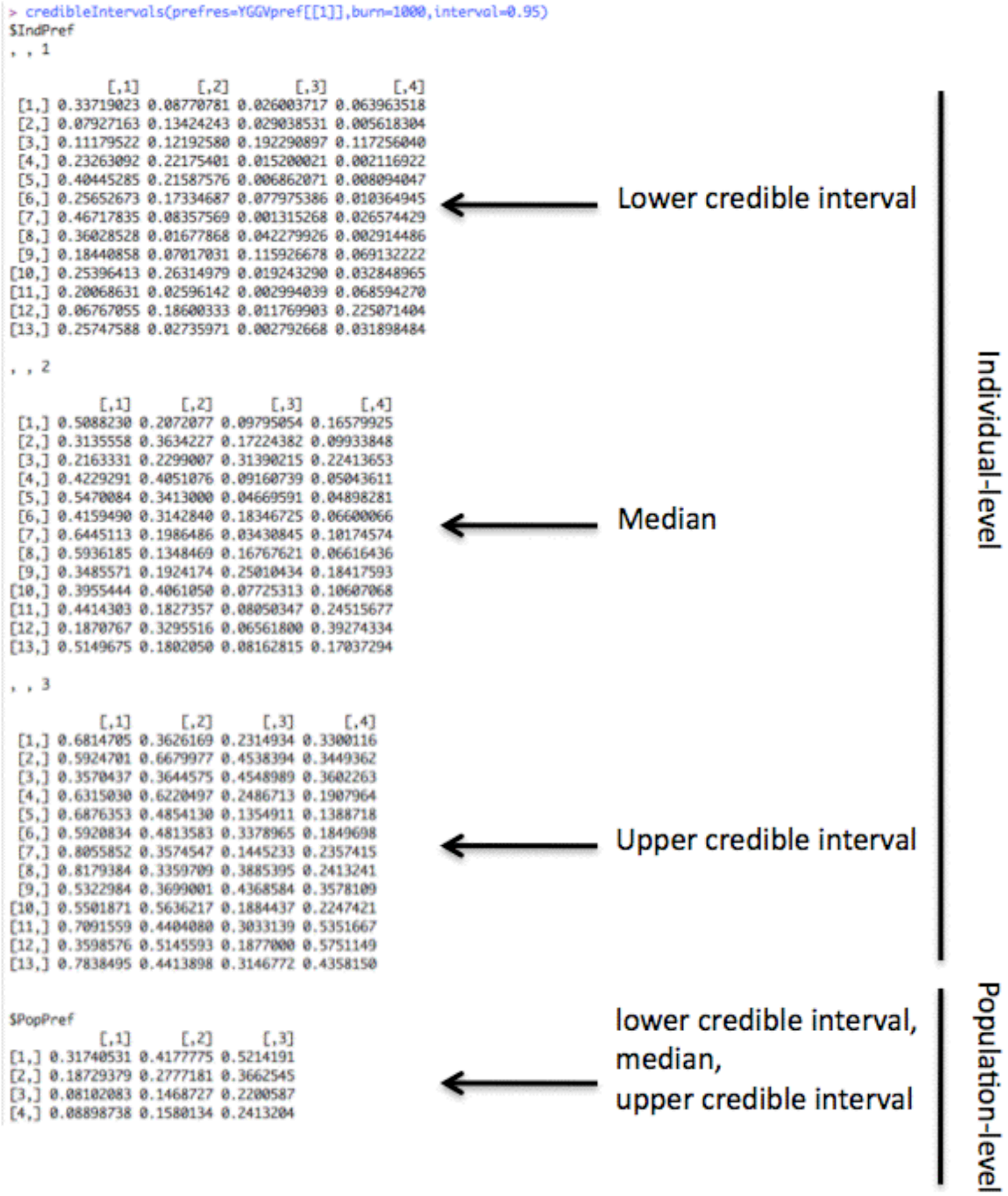

```
> credibleIntervals(prefres=YGGVpref[[1]],burn=1000,interval=0.95)
$IndPref
, , 1

            [,1]       [,2]        [,3]        [,4]
 [1,] 0.33719023 0.08770781 0.026003717 0.063963518
 [2,] 0.07927163 0.13424243 0.029038531 0.005618304
 [3,] 0.11179522 0.12192580 0.192290897 0.117256040
 [4,] 0.23263092 0.22175401 0.015200021 0.002116922
 [5,] 0.40445285 0.21587576 0.006862071 0.008094047
 [6,] 0.25652673 0.17334687 0.077975386 0.010364945
 [7,] 0.46717835 0.08357569 0.001315268 0.026574429
 [8,] 0.36028528 0.01677868 0.042279926 0.002914486
 [9,] 0.18440858 0.07017031 0.115926678 0.069132222
[10,] 0.25396413 0.26314979 0.019243290 0.032848965
[11,] 0.20068631 0.02596142 0.002994039 0.068594270
[12,] 0.06767055 0.18600333 0.011769903 0.225071404
[13,] 0.25747588 0.02735971 0.002792668 0.031898484

, , 2

           [,1]      [,2]       [,3]       [,4]
 [1,] 0.5088230 0.2072077 0.09795054 0.16579925
 [2,] 0.3135558 0.3634227 0.17224382 0.09933848
 [3,] 0.2163331 0.2299007 0.31390215 0.22413653
 [4,] 0.4229291 0.4051076 0.09160739 0.05043611
 [5,] 0.5470084 0.3413000 0.04669591 0.04898281
 [6,] 0.4159490 0.3142840 0.18346725 0.06600066
 [7,] 0.6445113 0.1986486 0.03430845 0.10174574
 [8,] 0.5936185 0.1348469 0.16767621 0.06616436
 [9,] 0.3485571 0.1924174 0.25010434 0.18417593
[10,] 0.3955444 0.4061050 0.07725313 0.10607068
[11,] 0.4414303 0.1827357 0.08050347 0.24515677
[12,] 0.1870767 0.3295516 0.06561800 0.39274334
[13,] 0.5149675 0.1802050 0.08162815 0.17037294

, , 3

           [,1]      [,2]      [,3]      [,4]
 [1,] 0.6814705 0.3626169 0.2314934 0.3300116
 [2,] 0.5924701 0.6679977 0.4538394 0.3449362
 [3,] 0.3570437 0.3644575 0.4548989 0.3602263
 [4,] 0.6315030 0.6220497 0.2486713 0.1907964
 [5,] 0.6876353 0.4854130 0.1354911 0.1388718
 [6,] 0.5920834 0.4813583 0.3378965 0.1849698
 [7,] 0.8055852 0.3574547 0.1445233 0.2357415
 [8,] 0.8179384 0.3359709 0.3885395 0.2413241
 [9,] 0.5322984 0.3699001 0.4368584 0.3578109
[10,] 0.5501871 0.5636217 0.1884437 0.2247421
[11,] 0.7091559 0.4404080 0.3033139 0.5351667
[12,] 0.3598576 0.5145593 0.1877000 0.5751149
[13,] 0.7838495 0.4413898 0.3146772 0.4358150


$PopPref
           [,1]      [,2]      [,3]
[1,] 0.31740531 0.4177775 0.5214191
[2,] 0.18729379 0.2777181 0.3662545
[3,] 0.08102083 0.1468727 0.2200587
[4,] 0.08898738 0.1580134 0.2413204
```

The results can be plotted using the function **prefPlot**.

```
prefPlot(YGGVpref[[1]],burn=1000,ymax=15)
```

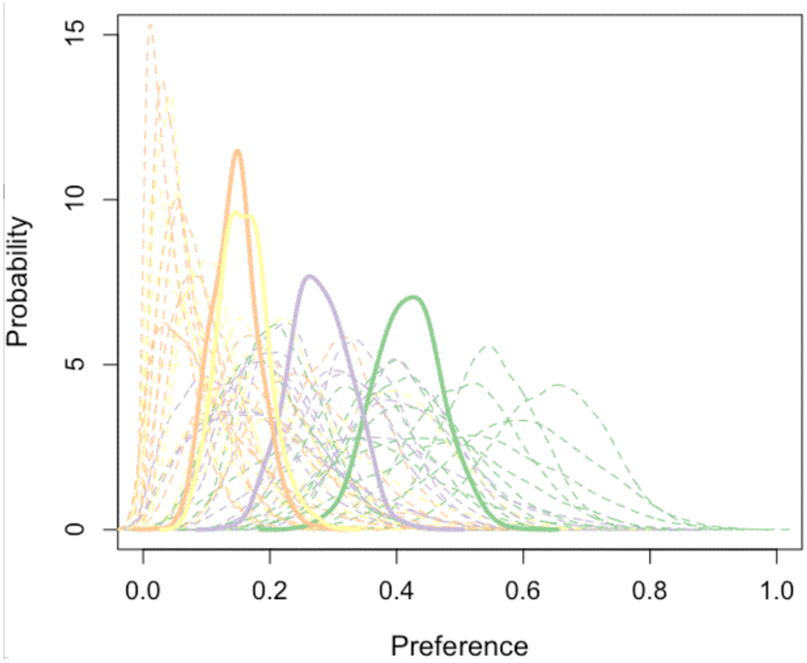

The solid lines show the estimates for the population-level preference for each plant and the dotted lines show the preference parameter estimates for each plant for each individual.

Pairwise comparisons:

Pairwise comparisons for population-level preference based upon the rankings of preference parameters for each step in the post burnin MCMC can be calculated using the function **pairwiseProb**.

```
pairwiseProb(YGGVpref[[1]],burn=1000)
```

```
> pairwiseProb(YGGVpref[[1]],burn=1000)
       [,1]   [,2]  [,3]   [,4]
[1,] 0.0000 0.9435 1.000 0.9995
[2,] 0.0565 0.0000 0.974 0.9615
[3,] 0.0000 0.0260 0.000 0.4210
[4,] 0.0005 0.0385 0.579 0.0000
```

These can be interpreted as one-tailed "post-hoc" tests. Thus, the probability that plant 1 is preferred over plant 2 is 0.0565. Likewise, the probability that plant 2 is preferred over plant 1 is 0.9435.

Comparing models:

Deviance Information Criterion (DIC) can be used to choose the best-fit model for the data. One can ask whether it is best to model the data where the parameter value describing preference is constrained to be the same. This is accomplished by using the argument `constrain=TRUE` in the function **bayesPref**.

Restricting our examination to only one population, here Yuba Gap, we can compare a model where the preferences are the same among all host plants, or whether they are best modeled as having different preference parameters.

```
YG<-YGGV[1:13,] # Create an object that only includes Yuba Gap, or population 1.
YGpref<-bayesPref(pData=YG,mcmcL=5000,pops=TRUE,dicburn=1000,dirvar=2)
YGprefconstrained<-bayesPref(pData=YG,mcmcL=5000,pops=TRUE,dicburn=1000,dirvar=2,constrain=TRUE)
```

YGpref DIC: 86.75607
YGprefconstrained DIC: 102.1706

The model used to generate `YGpref`, the unconstrained model, is more favorable compared to the model used to generate `YGprefconstrained`, the constrained model.

Similarly, whether or not populations should be modeled as having separate preferences, vs. a common, constrained preference can be examined using the argument `constrainP` in the **bayesPref** function.

```
YGGVpopconstrain<-bayesPref(pData=YGGV,mcmcL=5000,pops=TRUE,dicburn=1000,dirvar=2,constrainP=c(1,1))
```

Here, `constrainP=c(1,1)` sets the first and second population as being modeled as a single group.

Constrained DIC: 156.6923
Unconstrained DIC: 154.8371

Here, the constrained model and the unconstrained model are in the same family of "best models". In the end, the user must decide on the best model for the data. If the parameter estimates and CI are the goal (perhaps to be passed on to other analyses), the populations should be modeled separately.

Note: When multiple populations are involved, any groupings can be made. For example, if there were 4 populations, `constrainP=c(1,1,2,3)` would group populations 1 and 2, and allow populations 3 and 4 to be modeled as having there own preference.
